# Personnel Monitoring in Shipboard Surveillance Using Improved Multi-Object Detection and Tracking Algorithm

**DOI:** 10.3390/s24175756

**Published:** 2024-09-04

**Authors:** Yiming Li, Bin Zhang, Yichen Liu, Huibing Wang, Shibo Zhang

**Affiliations:** 1 Marine Engineering College, Dalian Maritime University, Dalian 116026, China; ymli@dlmu.edu.cn (Y.L.); 18739176570@163.com (Y.L.); peterzhang@dlmu.edu.cn (S.Z.); 2 Information Science and Technology College, Dalian Maritime University, Dalian 116026, China; huibing.wang@dlmu.edu.cn

**Keywords:** shipboard surveillance, personnel on ship, ship anti-intrusion, object detection, multi-object tracking

## Abstract

Detecting and tracking personnel onboard is an important measure to prevent ships from being invaded by outsiders and ensure ship security. Ships are characterized by more cabins, numerous equipment, and dense personnel, so there are problems such as unpredictable personnel trajectories, frequent occlusions, and many small targets, which lead to the poor performance of existing multi-target-tracking algorithms on shipboard surveillance videos. This study conducts research in the context of onboard surveillance and proposes a multi-object detection and tracking algorithm for anti-intrusion on ships. First, this study designs the BR-YOLO network to provide high-quality object-detection results for the tracking algorithm. The shallow layers of its backbone network use the BiFormer module to capture dependencies between distant objects and reduce information loss. Second, the improved C2f module is used in the deep layer of BR-YOLO to introduce the RepGhost structure to achieve model lightweighting through reparameterization. Then, the Part OSNet network is proposed, which uses different pooling branches to focus on multi-scale features, including part-level features, thereby obtaining strong Re-ID feature representations and providing richer appearance information for personnel tracking. Finally, by integrating the appearance information for association matching, the tracking trajectory is generated in Tracking-By-Detection mode and validated on the self-constructed shipboard surveillance dataset. The experimental results show that the algorithm in this paper is effective in shipboard surveillance. Compared with the present mainstream algorithms, the MOTA, HOTP, and IDF1 are enhanced by about 10 percentage points, the MOTP is enhanced by about 7 percentage points, and IDs are also significantly reduced, which is of great practical significance for the prevention of intrusion by ship personnel.

## 1. Introduction

The safe navigation of vessels as well as safe berthing is an important guarantee for the development of the marine economy [1]. But boarding theft, operation violations by external construction workers, trespassing by unknown persons, etc., may cause safety accidents or property damage in vessel operation. Therefore, it is crucial to prevent intrusions on ships. Ship anti-intrusion contains a series of links such as personnel tracking, face recognition, danger alarms, etc. Personnel tracking is seen as a useful method for ship anti-intrusion. Accurate and rapid personnel tracking helps to carry out subsequent operations in a timely manner, thereby enhancing the effectiveness of anti-intrusion measures on ships. The use of shipboard surveillance video to detect and track people, and thus achieve the anti-intrusion of ship personnel, is of great significance in improving the safe operation of ships [2].

Deep learning has shown an excellent performance in both object detection and multi-object tracking, and it is the mainstream technique in this field [3]. Contemporary multi-object-tracking algorithms can be categorized into two groups: Tracking By Detection (TBD) and Joint Detection and Tracking (JDT) [4]. TBD has been a hot research topic in recent years, which is based on the principle of object detection for each frame in the video stream and then the object association between neighboring frames, i.e., tracking using the results of object detection [5]. JDT, on the other hand, uses the same network for the detection and embedding of appearance information, which is faster but often requires common parameters to be set for these two different tasks, and thus TBD performs better in terms of accuracy [6]. Typical TBD algorithms are SORT, DeepSORT, Deep OC-SORT, etc.

The TBD tracking algorithm relies on the detector’s object-detection results, so a well-performing object-detection algorithm can improve the overall tracking effectiveness [7,8,9]. The deep-learning-based YOLO algorithm is designed with a one-stage structure, which generates a bounding box and a confidence score on the whole image to achieve fast target localization and category prediction [10,11]. The YOLO algorithm has been rapidly iterated in recent years, and several versions have been developed. YOLOv4 [12] and YOLOv5 have been applied in many fields such as industry and medicine, and YOLOv8 developed by the Ultralytics team is one of the most advanced object-detection algorithms. Some scholars have already studied the YOLO algorithm in human body detection in recent years. The power of YOLOv4 is its ability to detect multiple objects in one cell by joint prediction [13], and Score-NMS is used to improve the YOLOv4 accuracy and speed in crowded group detection. The fusion model of nonlinear filtering and YOLOv5 was proposed [14], providing a superior solution for moving-object detection in surveillance images. Currently, there are relatively few studies of YOLOv8 applied to human detection under surveillance video.

In terms of TBD multi-object-tracking algorithms, the SORT algorithm [15] is proposed, which uses Faster R-CNN as a detector and matches the object-detection results with the prediction results of the Kalman filter through the Hungarian algorithm, thus realizing multi-object tracking. SORT provides a right direction for the research of TBD algorithms and lays the foundation for a series of SORT algorithms. It is found that occlusion between pedestrians often occurs in surveillance camera scenarios [16], which causes the SORT algorithm to return a relatively high number of identity switches. To address this problem, Wojke’s team proposed the DeepSORT algorithm, which introduces appearance information on top of the SORT algorithm, which is extracted by an offline-trained CNN network. Such CNN networks are called re-identification (Re-ID) networks. Observation-Centric SORT (OC-SORT) is proposed based on SORT as an alternative to SORT’s estimation-centric version [17], which has an excellent performance under occlusion and nonlinear motion and is more real time. Deep OC-SORT [18] is proposed, which uses the Re-ID network on top of OC-SORT to integrate appearance matching into the OC-SORT algorithm, and the tracking performance reaches new heights.

The above studies have shown that combining Re-ID networks is an effective means to improve the problem of pedestrian occlusion and identity switches in surveillance camera scenarios. The algorithm of the TBD paradigm uses an object-detection model as a detector to detect a pedestrian target, the target ID is determined by matching it with the prediction of the Kalman filter, and the Re-ID network is responsible for extracting the appearance features to introduce the appearance information. Therefore, when Re-ID networks are utilized in the algorithms of the TBD paradigm, instead of using their detection, matching, and ranking functions, the focus is on their feature-extraction capabilities [19]. In order to improve the effect of multi-object tracking, the selection of the Re-ID model has also become a hot research topic. ResNet [20] is proposed based on residual structure design, which is powerful in feature extraction and is one of the commonly used Re-ID networks today. OSNet [21] is proposed using the residual idea proposed by He [20] to fuse multi-scale features using a unified aggregation gate (UAG). OSNet is designed for the Re-ID task by solving the omni-scale feature-learning problem to accomplish the Re-ID task quickly and accurately. In recent years, some scholars have studied the performance of different detectors and trackers in multi-object tracking. Yuan et al. [22] used OSNet as the basis, extracted global information and fine-grained information by using a two-branch structure, improved the OSNet network, introduced a weighted BiFPN structure in YOLOv5, and finally combined the two to realize multi-object tracking.

However, there is a gap in the existing multi-object detection and tracking algorithms for shipboard surveillance scenarios. The characteristics of the scene include the following: the cabin space is narrow, with numerous equipment and dense personnel, which can easily cause occlusion; ships have more cabins, resulting in complex personnel movement trajectories, which are less regular compared to the trajectories of traffic scenarios; the monitoring perspective of the deck and corridor is deeper, resulting in more small targets in the image; and the hardware conditions of the vessel are limited, so the model volume should not be too large. All of these characteristics affect the detection and tracking performance. Aiming at the above problems, this study redesigns the network structure of YOLOv8 and OSNet and proposes a multi-object detection and tracking algorithm for the anti-intrusion prevention of the vessel’s personnel under the TBD paradigm. The major contributions include the following:

1. In response to the high density of people onboard, personnel occlusion, and the prevalence of small targets in locations such as decks, this study integrates YOLOv8 with a transformer by introducing the BiFormer module. BiFormer builds upon visual transformer architecture and achieves flexible sparse attention allocation through the design of bilevel routing. This approach enhances the detection capability of small and occluded targets while maintaining relatively low parameters.
2. To address the issue of model lightweight design, an improvement was made to the C2f block of YOLOv8. This enhancement involves adopting a lightweight design and introducing the RepGhost. By incorporating lightweight design principles and reparameterization techniques, the model maintains accuracy while improving inference speed.
3. To tackle the complexities of personnel movement trajectories and the challenges of occlusion, a refined approach introduces the Part OSNet network to extract richer appearance information. Part OSNet maintains both full-scale feature-extraction capabilities and a lightweight structure while leveraging four distinct pooling branches to extract part-level features. This refined framework combines motion-prediction insights from Kalman filters, detection data from an enhanced YOLOv8 model, and detailed appearance features from Part OSNet. The result is a sophisticated multi-object-tracking system.
4. This study independently constructs a Bohai Sea Ro-Ro Ship Dataset for multi-object detection and tracking. The dataset is taken from a shipboard surveillance video of the Bohai Sea Ro-Ro Ship, which contains different scenes such as the cabin, deck, and cockpit. A large number of comparison experiments show that the improved YOLOv8 significantly improves the speed and accuracy under the premise of parameter lightweighting. As for multi-object tracking, this study is tested on both the Bohai Sea Ro-Ro Ship Dataset and the MOT17 dataset, and the results show that this study’s method significantly improves the HOTA, MOTA, MOTP, IDF1, and IDSwitch.

The structure of this study for the remainder is as follows: Section 2 reviews existing YOLO algorithms, Deep OC-SORT, and OSNet. Section 3 elaborates on the proposed method improvements. Section 4 analyzes the experimental results. Section 5 concludes with a summary, emphasizing this study’s contributions.

## 2. Related Works

The core idea of the YOLO series is to extract features using convolutional neural networks and perform regression on the entire input image, achieving end-to-end object detection [23,24]. In recent years, the YOLO series has been updated multiple times, utilizing different neural network architectures, suitable loss functions, and IoU, leading to continuous performance breakthroughs. It has become the mainstream detector in the current TBD paradigm. YOLOv8 continues the core idea of the YOLO series by employing CSPDarknet as the backbone network for feature extraction and integrating feature fusion through a combination of FPN and PAN structures. However, YOLOv8 differs in its use of an Anchor-Free design, simplifying operations such as NMS during inference to enhance inference speed [25]. Additionally, it utilizes the C2f module with skip connections to extract richer gradient flow information. By employing a Decoupled Head in the head section, YOLOv8 conducts category prediction and localization prediction separately. Additionally, it utilizes classification loss and bounding box regression loss separately in the loss function. These modifications enable YOLOv8 to achieve an excellent balance between speed and accuracy in object-detection tasks, making it suitable as a detector in TBD algorithms.

SORT provides a valuable research direction for TBD algorithms. Building upon the principles of SORT, Deep OC-SORT associates object-detection results with predictions from Kalman filters, centered around observations, to address the trajectory interruptions caused by overlapping targets. Furthermore, it integrates appearance information through a Re-ID network to enhance the tracking performance in scenarios involving occlusion and nonlinear motion, all while ensuring real-time capability.

In TBD algorithms that incorporate appearance information, the performance of Re-ID networks is particularly crucial [26]. The foundational module of OSNet comprises multiple convolutional streams with different receptive field sizes, with each module capturing features at different scales [27]. This design is driven by the recognition that the key to Re-ID tasks lies in learning discriminative features. For pedestrian ID determination, it is essential to utilize both global features to ascertain the approximate target range and local features to discern different pedestrian IDs in detail [28]. OSNet also incorporates a structure known as the aggregation gate (AG), which dynamically adjusts weights based on different input images. This allows it to focus on specific scales or fuse features from different scales, dynamically achieving feature fusion across various scales and ultimately obtaining multi-scale feature maps. While learning multi-scale information, OSNet utilizes depth-wise separable convolution (DW Conv) to reduce network parameters. These characteristics enable OSNet to fully extract multi-scale information while maintaining a lightweight design.

## 3. Our Approach

**Figure 1:**
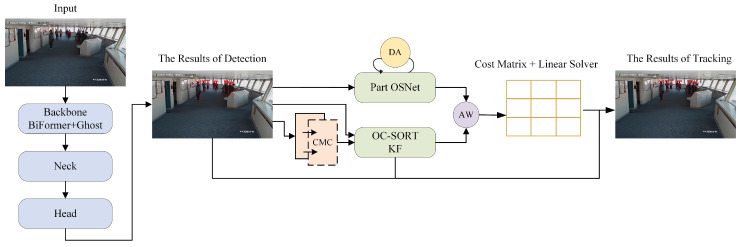
Structure of the tracking algorithm.

In this paper, a multi-object detection and tracking algorithm is proposed, which aims to detect and track the key people in shipboard surveillance images with high quality and then provide the basis for ship-intrusion prevention. The structure of the tracking algorithm is shown in Figure 1. Initially, each frame of the input image undergoes object detection using the enhanced YOLOv8 detector. The detection results are then input into the proposed Part OSNet to extract appearance information. Subsequently, the cost matrix is updated using the appearance information and motion prediction generated by the Kalman filter. Finally, the Hungarian algorithm is employed on the cost matrix to associate and match the object-detection results with the motion-prediction results, yielding the final trajectories. The overall workflow of Deep OC-SORT, including the Hungarian algorithm for association matching from the cost matrix, is retained in this study. This is because Deep OC-SORT effectively integrates the appearance information extracted by Part OSNet.

### 3.1. BR-YOLO

**Figure 2:**
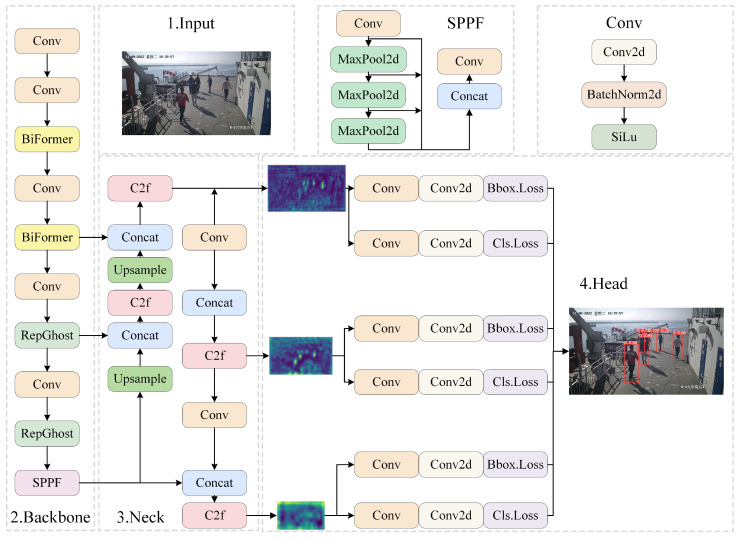
Improved YOLOv8 network architecture.

To improve detection quality in shipboard surveillance scenarios, this section addresses the issues of people onboard frequently obstructing each other, the presence of many small targets on the deck, and the requirement for the model to remain compact. The BR-YOLO algorithm is proposed, and its structure is shown in Figure 2. The algorithm retains the network structure of the Neck and Head from YOLOv8n. Instead of extensively utilizing the C2f module in the backbone network, BR-YOLO incorporates the BiFormer structure at shallow layers to enhance the feature-extraction capability while maintaining lower parameters. Additionally, improvements are made to the retained C2f module by introducing the RepGhost structure, ensuring accuracy is maintained while reducing computational complexity through reparameterization.

#### 3.1.1. BiFormer

The confined space within vessel cabins makes occlusion a common occurrence, especially during personnel activities. In shipboard surveillance scenarios, occlusion among pedestrians is more frequent compared to traffic scenes, and it often occurs earlier. Unlike pedestrians in traffic scenes, who typically walk in limited, fixed directions, occlusion among vessel personnel can happen at any time due to the diverse destinations they navigate toward. Moreover, the presence of numerous equipment and complex pipelines onboard increases the likelihood of occlusion events. Additionally, monitoring angles in areas like decks and corridors are often deep, leading to a higher density of small targets. This can result in decreased detection accuracy or even missed detections, thereby impacting both detection and tracking effectiveness.

**Figure 3:**
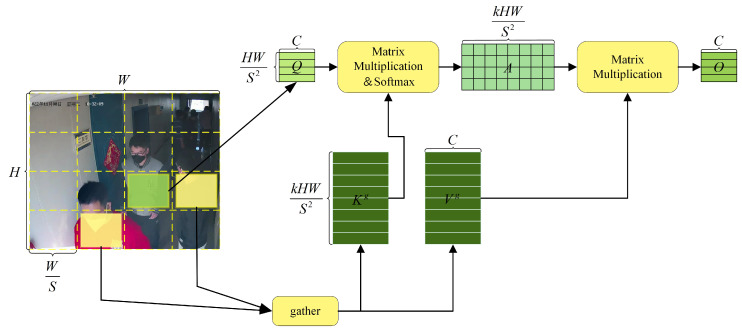
BRA structure.

To address the aforementioned challenges, this study starts with the detector and introduces the BiFormer structure in the shallow layers of the BR-YOLO backbone network. BiFormer is a visual transformer based on sparse attention mechanisms, with its core component being bilevel routing attention (BRA) [29]. The key idea of BRA is to filter out most irrelevant key–value pairs at a coarse region level so that only a small portion of routed regions remain, thus removing redundant information. Then, fine-grained attention is applied within the selected routed regions’ union. BRA restricts the scope of attention calculation, thereby improving performance while reducing computational complexity. The schematic diagram of BRA is illustrated in Figure 3.

**Figure 4:**
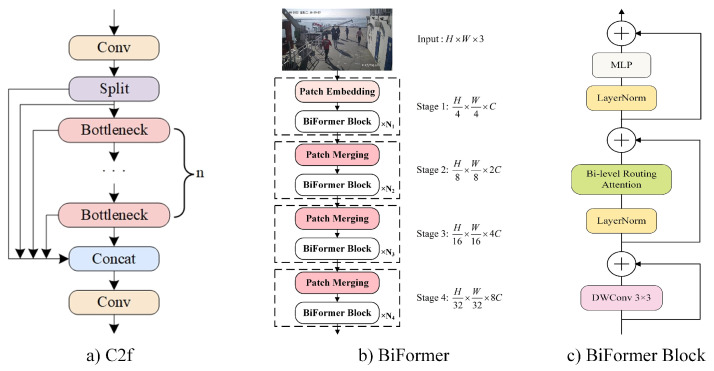
Network structure diagram of BiFormer and C2f.

BiFormer employs BRA as its core building block and adopts a four-layer pyramid structure, as illustrated in Figure 4. The network can be divided into four stages: Stage 1 uses overlapping image block embedding, while Stages 2 to 4 consist of image-fusion modules that reduce resolution, increase channel numbers, and utilize consecutive BiFormer blocks to transform the input data’s features. Each BiFormer block begins by applying depth-wise separable convolution to implicitly encode relative positional information. Next, the BRA module captures the bidirectional relative attention, followed by an MLP module for relationship modeling and positional embedding.

From the analysis above, it can be inferred that BiFormer, leveraging the structural characteristics of transformers, utilizes BRA for sparse sampling, replacing the downsampling of convolutional operations in convolutional neural networks (CNNs). CNNs extract features through convolutional layering, demonstrating stronger capabilities in extracting local features. However, they often lack long-distance connections for global image understanding, and downsampling operations in convolutional layers may lead to information loss. The C2f module in YOLOv8 is composed of numerous convolutional layers, embodying a typical CNN structure. For these reasons, this paper integrates the BiFormer module in the shallow layers and the C2f module in the deep layers of the BR-YOLO backbone network. Figure 4 illustrates the network architectures of BiFormer and C2f.

The BiFormer module is used in the shallow layers of the network because the sparse attention mechanism can effectively capture long-range inter-object dependencies, enhancing the extraction of global features and contributing to localization tasks. Moreover, the BiFormer module, devoid of convolutional operations, avoids the stacking of numerous convolutional layers throughout the backbone network, thereby alleviating issues related to information loss caused by downsampling. This approach is friendly to small object detection while fully preserving information in feature maps for deeper layers to utilize. Hence, the BiFormer module is more suitable for use in shallow layers. Conversely, the C2f module is employed in the deep layers due to its utilization of cross-layer connections. It adequately extracts deep semantic information through downsampling, enhancing its ability to capture local features, which is beneficial for category prediction. In shipboard surveillance videos, the confined space within cabins results in people being densely packed and frequently obstructing each other. Therefore, integrating the global information extracted by the BiFormer module through long-range dependencies with the deep information extracted by C2f through convolutional operations is more conducive to object detection in shipboard surveillance.

#### 3.1.2. RepGhost-C2f

Deep learning models with higher accuracy tend to have larger volumes and require more computational resources. The performance of existing object-detection and multi-object-tracking algorithms is often proportional to the model volume. Larger models require more stringent deployment conditions and higher-end hardware support. To address this issue, this study takes a lightweight approach and proposes the RepGhost-C2f module, integrated into the backbone network of BR-YOLO. This module reduces the model size of the detector, allowing more space for subsequent tracking processes. Simultaneously, it enhances the inference speed of BR-YOLO, aiming to promptly update detection states in the tracking algorithm and thereby further improving the tracking performance.

**Figure 5:**
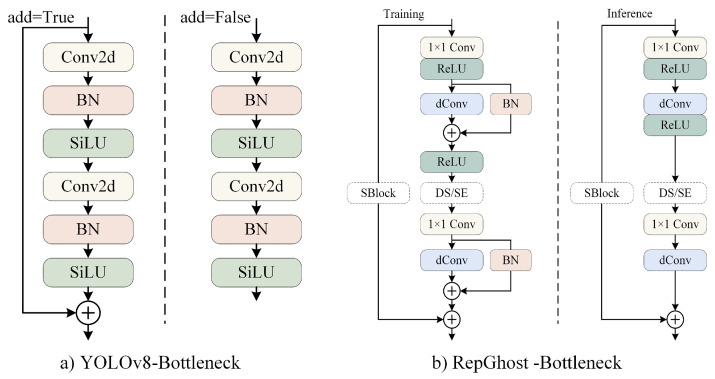
Comparison diagram of Bottleneck structure.

The core idea behind the RepGhost structure is to improve GhostNet through reparameterization to achieve implicit feature reuse [30]. GhostNet is a lightweight neural network that utilizes low-cost operations to generate a large number of feature maps, which are then concatenated with the original features to produce additional feature maps [31]. Concatenation operations indeed have a significant effect on reducing the model’s parameters and FLOPs. However, in practical applications, the efficiency of concatenation operations is much lower than that of addition operations, and they also incur higher computational costs on hardware [32]. Instead of inefficient concatenation operations, RepGhost adopts the more efficient addition operation to implicitly execute the information-fusion process, thus improving the inference speed. Another advantage of RepGhost is that the number of input channels is equal to the number of output channels during the inference stage, further saving inference time on hardware devices while maintaining the generation of different feature maps. The RepGhost Bottleneck is an improvement over the Ghost Bottleneck based on the RepGhost structure, directly replacing the Ghost module within it. The Bottleneck in YOLOv8n consists of two 3 × 3 convolution modules. Compared to the Bottleneck in YOLOv8n, structural reparameterization in the RepGhost Bottleneck reduces the inference process to just two branches, saving hardware memory costs and improving the inference speed. The structural diagrams of the two are illustrated in Figure 5. The RepGhost Bottleneck is highly concise during inference, consisting of only one direct connection module branch and a single-chain operation composed of 1 × 1 convolution, Depth-wise convolution, and ReLU. This has a significant impact on reducing model size and improving the inference speed. RepGhost Bottleneck also ensures feature fusion across different layers during training through reparameterization, maintaining the generation of different feature maps and thus preserving the feature-extraction capability of BR-YOLO.

**Figure 6:**
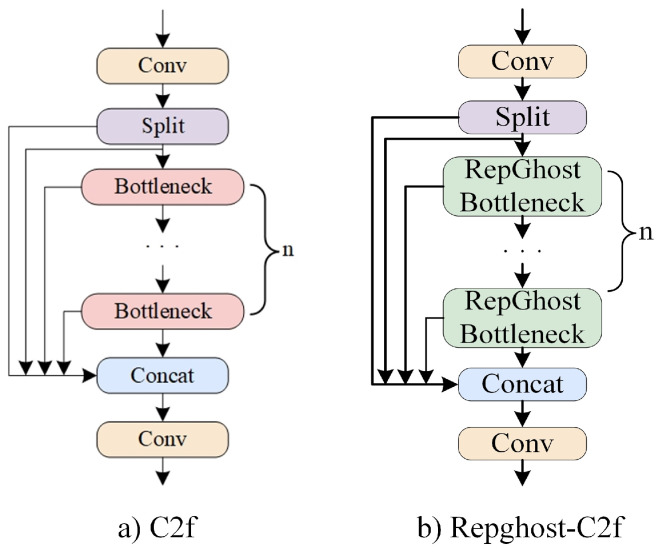
Comparison diagram of C2f structure.

This study uses the RepGhost concept to improve the C2f module, replacing the original Bottleneck module in C2f with the RepGhost Bottleneck and naming it RepGhost-C2f. It is utilized in the deep layers of the BR-YOLO backbone network, and its structure is depicted in Figure 6. RepGhost-C2f retains the core structure of C2f but inherits the characteristics of RepGhost, such as less parameters, simplified computations, and a faster inference speed, by introducing reparameterized lightweight structures. The improvements of RepGhost-C2f on the detector volume, detector inference speed, and multi-object-tracking performance for shipboard-intrusion prevention will be validated in Section 4.4 and Section 4.5.

### 3.2. OSNet Based on Part-Level Feature

**Figure 7:**
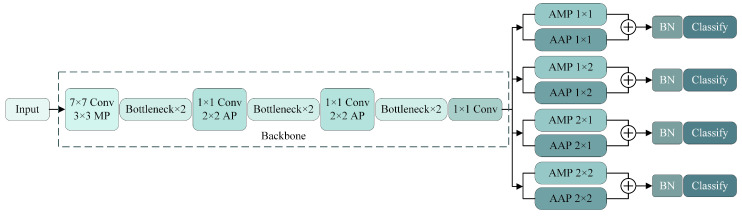
Part OSNet backbone network schematic.

Traffic surveillance is often deployed at street corners and intersections, where pedestrians typically move in fixed directions and have relatively predictable movement patterns. In shipboard surveillance, however, the personnel are mostly crew members and guests, who exhibit diverse movement directions, fast speeds, and complex and variable postures due to work requirements. This can lead to increased errors between the predicted and actual positions by the tracker, thereby reducing the tracking performance. To address the issues of mutual obstruction among personnel onboard and the complexity of their movement trajectories, this section introduces a pedestrian re-identification network named Part OSNet. This network is based on OSNet and focuses on part-level features. The part-level features efficiently contain a large amount of information, with a large amount of information and less redundant information, which can enhance the robustness of the model [33]. Therefore, Part OSNet can provide richer appearance information for multi-target-tracking algorithms. In situations with frequent occlusions, Part OSNet provides rich appearance information for ID confirmation. When the trajectory is complex, Kalman filter predictions may be inaccurate. At this point, the appearance information of Part OSNet can also improve the negative impact of inaccurate predictions. Figure 7 depicts the network architecture of Part OSNet, which generates feature maps containing rich global, local, and part-level features and updates network parameters through different loss functions.

**Figure 8:**
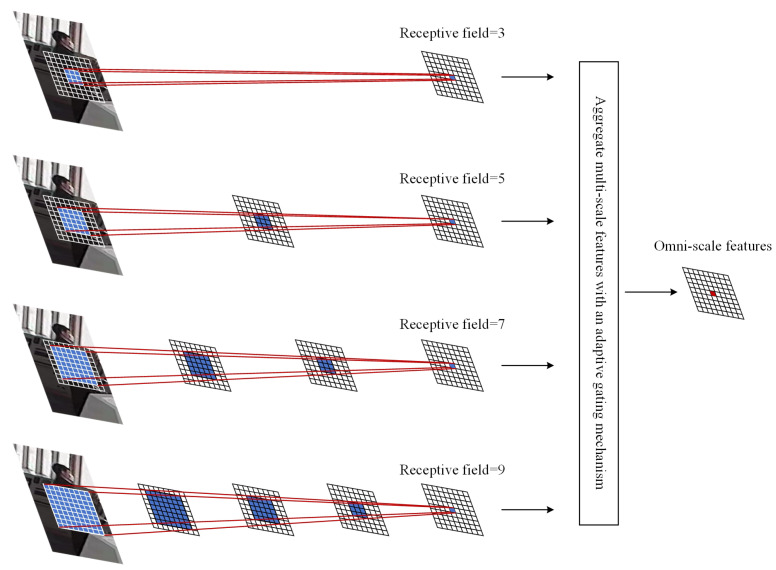
OSNet foundation building blocks schematic.

This study retains the backbone network of OSNet, keeping the structure identical to OSNet’s architecture up to the Global Average Pooling (GAP) layer. However, it removes the GAP layer and subsequent layers. The purpose of retaining the backbone network of OSNet is to preserve OSNet’s capability for full-scale feature extraction and its lightweight characteristics. OSNet learns comprehensive object-feature representations. Figure 8 illustrates the basic building block of OSNet, which consists of multiple convolutional streams with different receptive field sizes. Each stream focuses on features with scales determined by an exponentially increasing index to ensure the capture of different scales in each block. The resulting multi-scale feature maps are dynamically fused by the AG. The AG is a trainable small neural network with shared parameters among all streams, possessing many properties required for effective model training. By using a trainable AG, the generated channel weights depend on the input, enabling dynamic scale fusion.

After traversing all layers of the backbone network, Part OSNet employs four branches to process full-scale features. Each branch employs adaptive max pooling and adaptive average pooling, followed by combining the pooled results. Max pooling emphasizes the most prominent local regions, while average pooling focuses on background information. Their fusion contributes to extracting a more comprehensive set of feature information. The branches differ in their use of various adaptive pooling sizes to extract distinct features. The first branch utilizes a 1 × 1 adaptive pooling size to extract features from the entire full-scale feature map. The second branch employs a 1 × 2 size to pool horizontally. The third branch uses a 2 × 1 size for vertical pooling. In the fourth branch, the full-scale features are partitioned into four grids to extract part-level features.

**Figure 9:**
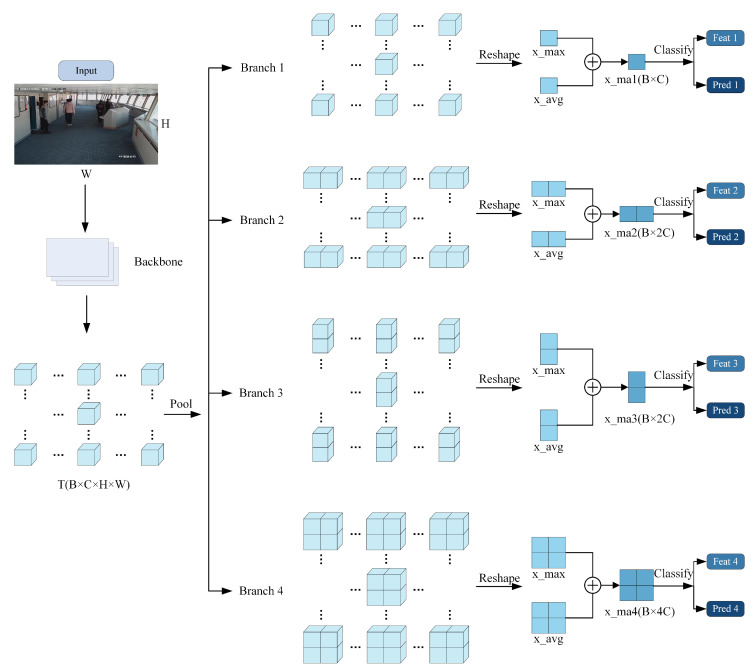
Operation flow of Part OSNet.

The feature-extraction process of Part OSNet is illustrated in Figure 9. After the input image passes through the OSNet-based backbone network, it yields a four-dimensional full-scale feature *T* with dimensions *B × C × H × W*. *T* is then fed into a multi-branch pooling network. In the first branch, *T* undergoes 1 × 1 AMP and AAP, followed by a reshape operation for dimension reduction, resulting in two-dimensional feature parameters x_max and x_avg with dimensions *B × C*. These are weighted to produce the output x_am1. Similarly, the second branch, after 1 × 2 AMP and AAP and reshaping, obtains two-dimensional feature parameters x_max2 and x_avg2 with dimensions *B × 2C*, which are weighted to produce x_am2. The third branch yields two-dimensional feature parameters x_am3 with dimensions *B × 2C*. The fourth branch produces two-dimensional feature parameters x_am4 with dimensions *B × 4C*. The two-dimensional feature parameters from the four branches are separately fed into classifiers. Bn layers normalize them, resulting in feat1, feat2, feat3, and feat4, along with prediction results Pred1, Pred2, Pred3, and Pred4. During testing, feat1, feat2, feat3, and feat4 are concatenated, and the concatenated feature map is used for prediction.

It is worth noting that during the training process, the ID prediction loss is evaluated using cross-entropy loss, while the feature-extraction loss for each branch is assessed using hard Triplet loss. The total loss is the weighted sum of these two components:

(1) $$\begin{array}{c} L=\lambda_{1}L_{1}+\lambda_{2}L_{2} \end{array}$$

In Equation (1), $\lambda_{1}$ and $\lambda_{2}$ are the weighting factors, $L_{1}$ is the feature-extraction loss, and $L_{2}$ is the ID prediction loss.

For the feature-extraction process in each branch, the loss is optimized using hard Triplet loss, which has been proven to be effective in Re-ID tasks [34]. It is expressed as Equation (2):

(2) $$\begin{array}{c} L_{\mathrm{th}\;}=\frac{1}{Q\times K}\sum_{a\in \;\mathrm{batch}\;}{\left(\max_{p\in A}d_{a,p}-\min_{n\in B}d_{a,n}+\alpha \right)}_{+} \end{array}$$

There, α is a manually set threshold parameter. The set of images with the same ID as a is *A*, and the remaining set of images with different IDs is *B*. For each batch, the number of randomly selected pedestrians is *Q*, and each pedestrian randomly selects *K* different pictures. TriHard loss calculates the Euclidean distance between each image and every other image in the batch in the feature space. Then, it selects the farthest positive sample *p* and the closest negative sample *n* to compute the Triplet loss. Typically, TriHard loss performs better than traditional Triplet loss:

(3) $$\begin{array}{c} s(x)=\ln(1+\exp(x)) \end{array}$$

In Equation (3), s(x) is an approximation function used to continuously pull the distances between objects of the same class closer.

For ID prediction, optimization is performed using cross-entropy loss, expressed as Equation (4):

(4) $$\begin{array}{c} L_{2}=-\sum y_{i}\log p_{i} \end{array}$$

There, $y_{i}$ is the true label and $p_{i}$ is the predicted probability by the model for the *i* class.

## 4. Experiment and Analysis

### 4.1. Experimental Platform and Parameter Settings

The configuration information for the experimental platform is as shown in Table 1. The operating system used is Windows 10, with an AMD EPYC 7542 32-Core Processor for the CPUand an NVIDIA GeForce RTX 4090 for the GPU. The system has 128 GB of memory. The development tool used is PyCharm 2021.3.3, with CUDA and cuDNN installed to aid GPU acceleration. The deep learning framework employed is PyTorch1.8.1.

The relevant information for the training parameters is shown in Table 2. The resolution of the input images is 640 × 640. The experiment is set for 500 training epochs, with a batch size of 16 and an initial learning rate of 0.002.

### 4.2. Evaluation Metrics

#### 4.2.1. Object-Detection Evaluation Metrics

To better quantify the effectiveness of the object-detection algorithm, this study evaluates it using five metrics: P (precision), R (recall), mAP (mean average precision), Param (parameters), and time. The formulas for the calculation are as Equations (5)–(8):

(5) $$\begin{array}{c} P=\frac{TP}{TP+FP} \end{array}$$

(6) $$\begin{array}{c} R=\frac{TP}{TP+FN} \end{array}$$

(7) $$\begin{array}{c} \mathrm{AP}=\int_{0}^{1}PRdR \end{array}$$

(8) $$\begin{array}{c} \mathrm{mAP}=\frac{1}{n}\sum_{i=1}^{n}AP_{i} \end{array}$$

There, TP represents the count of pedestrians where both the detection and the ground truth are true, FP represents the count of pedestrians where the detection is true but the ground truth is false, FN represents the count of pedestrians where the ground truth is true but the detection result is false, and TN represents the count of pedestrians where both the ground truth and the detection are false. n stands for the number of target categories (in this study, *n* = 1) and $AP_{i}$ represents the average precision (AP) for the target indexed by *i*. There, mAP0.5 calculates the mean of the average precision values for a recall value over 0 to 1 with an IoU threshold of 50%, and mAP0.5:0.95 has an IoU threshold between 50% and 95%.

#### 4.2.2. Multi-Object-Tracking Evaluation Metrics

In evaluating tracking algorithms, this study selects the HOTA (High-Order Tracking Accuracy), MOTA (Multi-Object-Tracking Accuracy), MOTP (Multi-Object-Tracking Precision), and IDF1 (Identification F1) as evaluation metrics. Table 3 presents the chosen evaluation metrics in this study along with their optimal evaluation trends. IDS represents the number of ID switches for the same pedestrian target. A lower value indicates the better tracking performance of the algorithm.

The HOTA introduces higher-dimensional tracking-accuracy metrics, offering a more comprehensive and evenly balanced assessment of the multi-object-tracking performance. The MOTA measures a tracker’s performance in both detecting targets and maintaining tracking. The MOTP gauges the localization precision of the detector. IDF1 assesses the stability of the tracker: higher values indicate that the algorithm can track targets more accurately and for longer durations.

The calculation formula for the HOTA is as shown in Equation (9), where DetA represents the detection accuracy score and AssA represents the association accuracy score. Here, *c* denotes a specific positive sample trajectory:

(9) $$\begin{array}{c} \mathrm{HOTA}=\sqrt{\mathrm{DetA}\cdot AssA}=\sqrt{\frac{\sum_{C\in TP}A\left(c\right)}{TP+FN+FP}} \end{array}$$

where A(c) represents the accuracy of the associated evaluation data, calculated as in Equation (10). For the TP set, TPA(c) refers to cases where the predicted ID and detection box are both *c*; FNA(c) refers to cases where the true value is *c* in the TP set, but the predicted ID is not *c*, and cases where the true value is *c* in the FN set; and FPA(c) refers to cases where the predicted ID is *c* in the TP set, but the true value is not *c*, and cases where the predicted ID is *c* in the FP set:

(10) $$\begin{array}{c} A\left(c\right)=\frac{\left|TPA(c)\right|}{\left|TPA(c)|+|FNA(c)|+|FPA(c)\right|} \end{array}$$

The MOTA represents the accuracy of multi-object tracking, where IDSW represents the number of times the target label ID switched during tracking in frame *t* and $g_{t}$ represents the number of targets observed at time *t*. The calculation is as shown in Equation (11):

(11) $$\begin{array}{c} \;\mathrm{MOTA}\;=1-\frac{\sum_{t}\left(FP+FN+IDSW_{t}\right)}{\sum_{t}g_{t}} \end{array}$$

The MOTP represents the precision of multi-object tracking and is used to quantify the localization accuracy of the detector. There, $d_{t}^{i}$ represents the distance between the given position and its paired hypothetical position in frame *t*, $c_{t}$ represents the number of matches between the targets and hypothesis positions in frame *t*, and *i* represents the current detection target. The calculation is as shown in Equation (12):

(12) $$\begin{array}{c} \mathrm{MOTP}=\frac{\sum_{i,t}d_{t}^{i}}{\sum_{t}c_{t}} \end{array}$$

IDF1 refers to the F1 score of target ID recognition within each target-bounding box, where IDTP represents the total number of targets correctly tracked with an unchanged ID, IDFP represents the total number of targets incorrectly tracked with an unchanged ID, and IDFN represents the total number of targets lost in tracking with an unchanged ID. The calculation is as shown in Equation (13):

(13) $$\begin{array}{c} \;\mathrm{IDF}\;1=\frac{2}{\frac{1}{IDP}+\frac{1}{IDR}}=\frac{2IDTP}{2IDTP+IDFP+IDFN} \end{array}$$

### 4.3. Dataset Construction

In this study, two datasets are constructed, one for training the object-detection model and another for validating the multi-object-tracking algorithm. Additionally, the Market1501 dataset is utilized to train the feature-extraction model.

**Figure 10:**
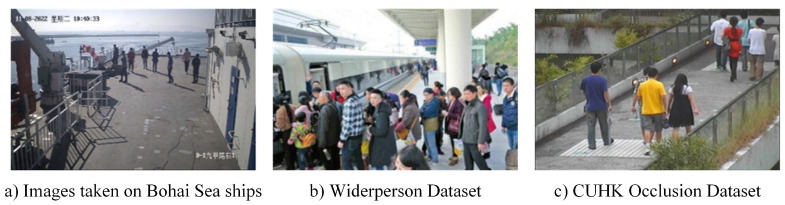
Example of autonomous datasets.

Since there is no publicly available dataset of onboard surveillance personnel on the Internet, this study obtains the shipboard surveillance data of the Bohai Sea Ro-Ro Ship and preprocesses these data to construct the dataset independently. The Bohai Sea Ro-Ro Ship Dataset was collected from real shipboard surveillance videos, where personnel trajectories are more complex and the space is more confined than in traffic scenarios. This makes it a more effective dataset for fully validating the algorithm proposed in this paper. In order to ensure the diversity of the object-detection dataset, the surveillance video is divided into two parts. In the first part, the cropped video clips are labeled using DarkLabel 2.4 with every 20 frames sampled and added to the object-detection dataset. The remaining surveillance video segments are used to validate the multi-object-tracking algorithm. In addition, due to the limited number of shipboard surveillance videos and the lack of personnel richness, this study, in order to enhance the robustness of the object-detection model and improve the model feature-extraction capability, adds the traffic scene pedestrian dataset, CUHK Occlusion Dataset, and WiderPerson Dataset to the dataset, as shown in Figure 10. Among them, the CUHK Occlusion Dataset is the mainstream pedestrian-detection dataset, shot on the campus of CUHK, with rich pedestrian features and diverse pedestrian situations, including occlusion and re-identification. The WiderPerson Dataset is a dense-crowd dataset with a large number of people overlapping and with diverse appearances, which is no longer confined to the traffic scenario and enriches the background, which can make up for the lack of diversity in the shipboard surveillance and the lack of diversity in traffic scenarios. These three parts together constitute the shipboard pedestrian object-detection dataset, which contains 10,678 images, and is divided into the training set (8542 images), the validation set (1068 images), and the test set (1068 images) according to the ratio of 8:1:1. It uses Labelimg for annotation, and the pedestrian class is named as 0.

In order to validate the multi-object-tracking algorithm, the Bohai Sea Vessel Dataset is used as the test data. To compare the tracking capabilities of the model across different scenarios, eight videos were selected. After multiple people annotated the data using the DarkLabel software, testing was conducted to prevent labeling errors from affecting the model’s performance [35]. The records are numbered from 01 to 08, as detailed in Table 4.

### 4.4. Object-Detection Experiments

#### 4.4.1. Comparison of Different Attention Mechanisms

**Figure 11:**
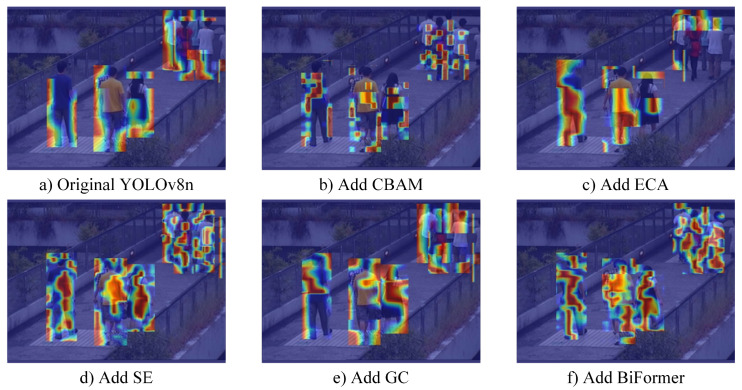
Results of Grad-CAM heat map visualization.

For the purpose of validating the effectiveness of BiFormer, this study incorporated four additional attention mechanisms into the backbone network for comparison. The results are shown in Table 5. From Table 5, it can be observed that BiFormer achieves the highest increase in accuracy with the least parameter increments. This is due to the fact that BiFormer achieves sparse attention through a two-layer routing mechanism, which enables the model to focus more on relevant channel information. A visual comparison of the experimental results using the Grad-CAM method, as depicted in Figure 11, reveals that models employing BiFormer exhibit more accurate coverage of all pedestrian targets with their Grad-CAM masks, indicating a more precise focus. In other words, the BiFormer module can better capture target-related features, thereby enhancing the detection performance.

#### 4.4.2. Quantitative Comparison Experiments

**Figure 12:**
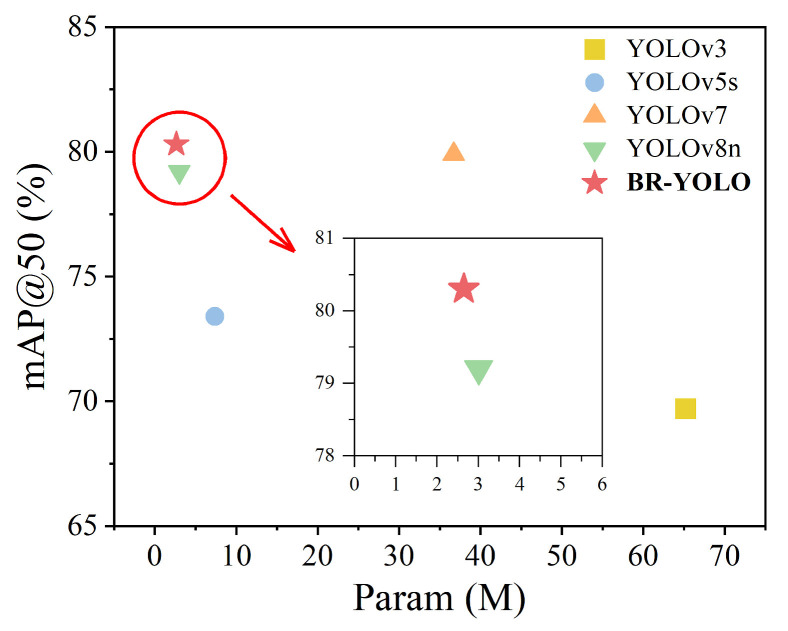
Performance comparison of object-detection algorithm.

To validate the detection performance of the model improved upon YOLOv8n as proposed in this study, we trained YOLOv3, YOLOv5s, YOLOv7, and the original YOLOv8n model using the same dataset. Subsequently, we evaluated the performance of these models using the test dataset. The experimental results are elegantly showcased in Table 6 and Figure 12.

As shown in Table 6, compared to YOLOv3 and YOLOv5s, BR-YOLO demonstrates substantial improvements in precision, recall, mAP0.5, and mAP0.5:0.95. Moreover, there are notable optimizations in terms of the parameters and average detection time. While BR-YOLO exhibits slightly lower precision and recall compared to YOLOv7, its accuracy is marginally higher, and its parameters are only 7.21% of that of YOLOv7, with a detection speed twice as fast, making it more suitable for deployment in maritime scenarios. From the enlarged image in Figure 12, it can be seen that BR-YOLO significantly improved. Compared to the original YOLOv8n model, the proposed model improves the precision, mAP0.5, and mAP0.5:0.95 by 0.7, 1.1, and 1.2 percentage points, respectively. The recall and average detection time remain the same, while the number of parameters is reduced by 12%.

#### 4.4.3. Ablation Experiments of Object Detection

To analyze the effects of the BiFormer module and RepGhost module, this study conducts ablation experiments on the improvement methods outlined in Section 3.1.1 and Section 3.1.2. In these experiments, four groups are tested that correspond to the original YOLOv8n, the application of only the BiFormer, the application of only the RepGhost-C2f, and the combined usage of both structures in BR-YOLO. Each experiment is conducted on the shipboard surveillance dataset, using the same training set and training parameters. The experimental results are presented in Table 7, where “✓” indicates the utilization of the method in the network while “-” denotes the absence of that method.

As shown in Table 7, Experiment 1 presents the training results of the original YOLOv8n, serving as the baseline for this study. Experiment 2 applies BiFormer within YOLOv8n, resulting in a noticeable enhancement in accuracy compared to Experiment 1. Specifically, precision, mAP0.5, and mAP0.95 increased by 1.2, 1.3, and 1.2 percentage points, respectively, albeit with increases in the parameters and detection time. In Experiment 3, RepGhost-C2f is employed within YOLOv8n, resulting in a significant reduction in the parameters and a notable improvement in the detection speed with minimal impact on accuracy. Experiment 4 introduces BR-YOLO, as proposed in this study. Compared to the original model, BR-YOLO demonstrates a reduction in the parameters of approximately 12%. While maintaining the same speed, it significantly enhances the detection accuracy, with increases of 0.7, 1.1, and 1.2 percentage points in the precision, mAP0.5, and mAP0.5:0.95, respectively. Thus, the shallow BiFormer block and the RepGhost-C2f block proposed play crucial roles in both improving the object-detection performance and lightweighting the model.

#### 4.4.4. Qualitative Comparison Experiments

**Figure 13:**
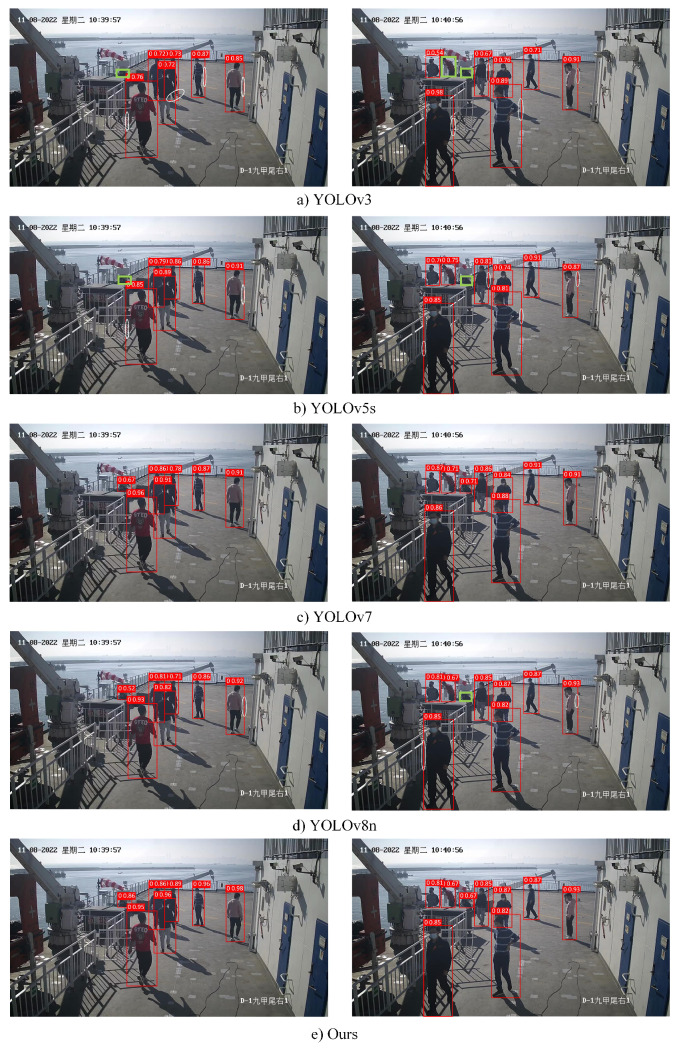
Object Detection comparison experiments (green box in the figure indicates a missed target, white circle circled for redundant background).

Figure 13 visually illustrates the performance of the proposed model in target detection. YOLOv3, YOLOv5, and YOLOv8n all exhibit missed detections on small targets, as shown in the green dashed boxes in Figure 13a,b,d. However, the improved model introduced in this paper incorporates the Biformer structure, enhancing its ability to detect small targets without any missed or false detections, thereby demonstrating a superior detection performance. Compared to the original YOLOv8 model, the improved model provides more accurate detection boxes with a less redundant background within the boxes.

In summary, the model proposed exhibits the highest precision, mAP0.5, and mAP0.5:0.95 among the four models, along with the least parameters and shortest detection time. These results indicate that the improved YOLOv8 model proposed possesses certain advantages in accurately identifying pedestrians in maritime scenarios.

### 4.5. Tracking Algorithms Experiments

#### 4.5.1. Quantitative Comparison Experiments

This subsection verifies the effectiveness of the multi-object-tracking algorithm proposed by comparing the current mainstream tracking algorithms. The compared algorithms include BoT-SORT [36], OC-SORT, StrongSORT [37], ByteTrack [38], and Deep OC-SORT. In the experiments of this subsection, the detectors employed by each group of algorithms use BR-YOLO proposed above, and the Re-ID networks used in the trackers are the original OSNet. Ours is the multi-object-tracking algorithm proposed in Section 3 of this study.

**Figure 14:**
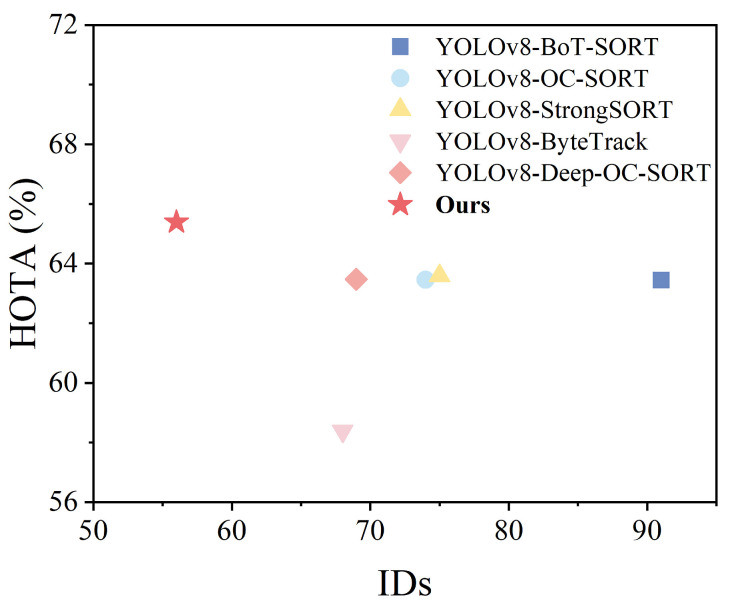
Performance comparison of tracking algorithm on the Bohai Sea Ro-Ro Ship Dataset.

**Figure 15:**
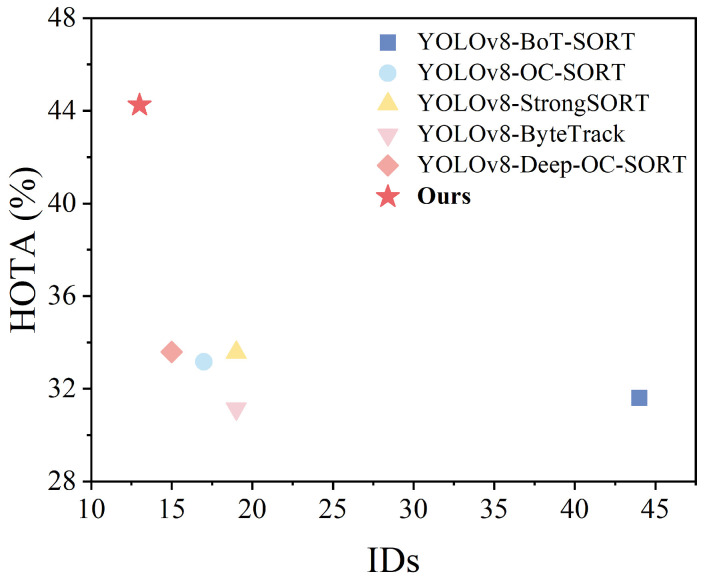
Performance comparison of tracking algorithm on MOT17.

Table 8 shows the performance of the popular multi-object-tracking algorithms on the Bohai Sea Ro-Ro Ship Dataset, and the algorithms include BoT-SORT, OC-SORT, StrongSORT, ByteTrack, and Deep OC-SORT. In this paper, we also carried out complementary experiments on the MOT17 dataset under the traffic scenario, and the experimental results are shown in Table 9. As can be seen from Figure 14 and Figure 15, the Deep OC-SORT performance is the best in both the Bohai Sea Ro-Ro Ship Dataset and the MOT17 dataset. Based on the above experimental analysis, DeepOC-SORT is selected in this paper as the basic framework of the shipboard multi-object-tracking algorithm.

Overall, compared to the MOT17 dataset, the metrics of the algorithms in the validation of the shipboard pedestrian dataset have lower values. The reason for analyzing the slightly lower MOTP is that the people onboard represent more common traffic scenarios, which are characterized by numerous movements, faster moving speeds, and complex and variable behavioral postures, and the error between the tracker’s predicted position and the actual position during pedestrian tracking increases, resulting in an inaccurate position estimation. Moreover, the small space and the large number of various equipment onboard lead to a serious problem of personnel occlusion, which is easy to result in ID switching, and it is difficult for the tracking system to correctly recognize the correlation relationship between targets, which in turn reduces the HOTA and MOTA scores of each algorithm. In surveillance videos, frequent occlusions among personnel significantly impact long-term tracking, resulting in a relatively low IDF1 score. The relatively fewer ID switches compared to traffic scenarios are attributed to the lower number of personnel onboard. Therefore, pedestrian-tracking environments in maritime surveillance are inherently more complex and challenging. The multi-object-tracking algorithm proposed for vessel intrusion detection integrates rich appearance information from Part OSNet. The results from both the BSV dataset and the MOT17 dataset demonstrate significant advancements across all metrics, providing strong evidence of the superiority of the algorithm proposed.

#### 4.5.2. Ablation Experiments of Tracking

The experiments in this subsection are all conducted on the Bohai Sea Ro-Ro Ship Dataset using Deep OC-SORT as the base framework of the tracking algorithm. As shown in Table 10, Experiment 1 uses the original YOLOv8 as the detector and the original OSNet as the Re-ID network, which is used as the baseline for the experiments in this section. Experiment 2 uses BR-YOLO as the detector and improves all four metrics significantly except for the IDs. Experiment 3 uses Part OSNet as the Re-ID network, which makes all the indexes better, and the enhancement is more significant compared to Experiment 1. This enhancement is more pronounced because Part OSNet not only extracts full-scale features but also captures part-level features, thereby notably addressing issues related to mutual occlusion among personnel on vessels and the complexity of their movement trajectories, which would otherwise affect the tracking performance. Test 4 shows that the improved two models are all put into Deep OC-SORT, and compared with the baseline, all the indexes are greatly improved, among which the HOTA, MOTA, MOTP, and IDF1 are the most significant, with an improvement of 10.66, 9.65, 6.62, and 11.4 percentage points, respectively. Taken together, it is evident that both the improved BR-YOLO and Part OSNet contribute significantly to the tracking algorithms individually. However, their combined usage yields even more pronounced improvements.

#### 4.5.3. Qualitative Comparison Experiments

**Figure 16:**
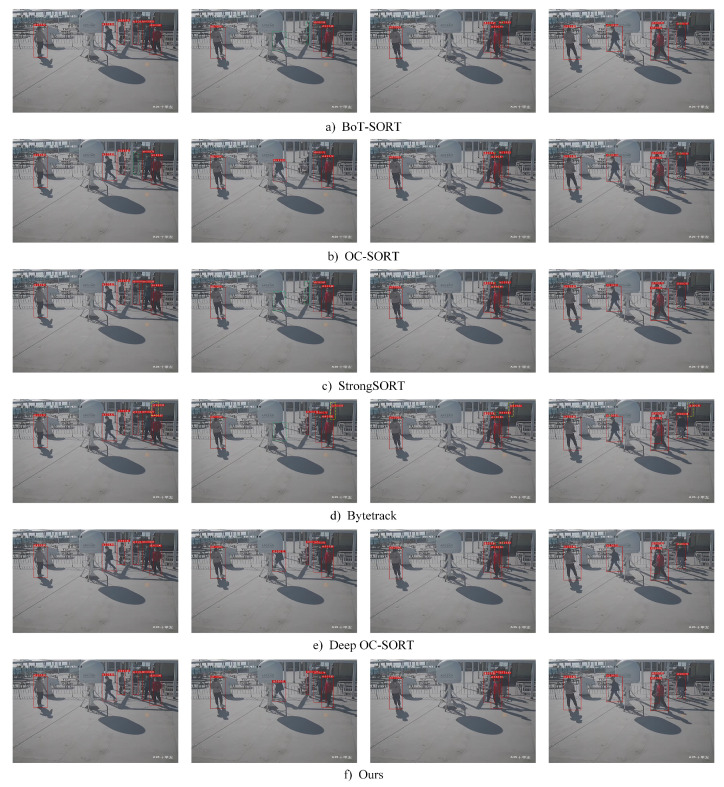
Results of multi-object-tracking algorithms (the green dashed line indicates a missed target, the circle indicates an ID error or skip, the blue dashed line indicates an incorrectly tracked target, and the yellow box indicates a misdetected target).

The BoT-SORT, OC-SORT, StrongSORT, ByteTrack, Deep OC-SORT, and the improved algorithm of this study in the visualization tracking results of Video 04 are shown in Figure 16. Video 04 was shot on the deck, in which there are some disturbing factors, such as columns, shipboard facilities, and mutual occlusions between characters. Compared with the Figure 16f results, the first five algorithms have missed detection (e.g., the targets in the green dashed frames in Figure 16a–e). However, our algorithm does not miss tracking targets and still has a good detection and tracking ability for targets with high occlusion, such as in the Figure 16f experiment, in which the target with an ID of six can still be accurately detected and tracked in frames 123 and 133 despite the presence of a large occlusion. Our algorithm is able to achieve the comprehensive detection and tracking of all targets. Due to the introduction of Part OSNet, it can be visualized that the ID jumps are reduced in the Figure 16f experiment.

In summary, our algorithm has a superior performance for shipboard pedestrian tracking compared with other multi-object-tracking methods and provides good technical support for shipboard safety management by realizing good multi-object tracking for shipboard pedestrians.

## 5. Conclusions

In this paper, a multi-object detection and tracking algorithm for shipboard surveillance is designed to improve the poor quality of existing algorithms in shipboard surveillance scenarios. The specific conclusions are as follows:

1. A multi-object detection and tracking dataset is constructed for shipboard surveillance scenarios. This dataset is derived from surveillance videos in the Bohai Sea Ro-Ro Ship, serving as both training and testing data for the models and providing essential data support for the research conducted.
2. This study meticulously examines the constraints of existing methodologies in shipboard surveillance settings and introduces targeted enhancements. For object detection, BR-YOLO is proposed as a detector for multi-object tracking. In response to the problem of dense population density and severe occlusion in the video images of shipboard surveillance, within BR-YOLO, the BiFormer module in its shallow layers aims to amplify the detection capabilities, particularly for small and densely packed objects. Moreover, the RepGhost-C2f module is introduced to optimize model parameters and maintain the accuracy and speed of reasoning to make the model easy to deploy on ships. In order to improve the negative effects of masking and the complexity of the movement trajectories of personnel on ships, the Part OSNet network is presented. This network employs four distinct pooling branches to extract features across various scales, thereby integrating a more comprehensive range of appearance information.
3. In comparative experiments conducted on the Bohai Sea Ro-Ro Ship Dataset, the proposed approach demonstrates superiority over existing mainstream methods. In terms of object detection, compared with YOLOv8, the precision, mAP0.5, and mAP0.5:0.95 of BR-YOLO increased by 0.7, 1.1, and 1.2 percentage points, respectively, and the parameters decreased by 12%. In terms of tracking, compared with the original algorithm, the method proposed in this article improved the HOTA, MOTA, MOTP, and IDF1 by 10.66, 9.65, 6.42, and 11.4 percentage points, respectively, and reduced IDs by 13.3%. Moreover, the method also shows significant improvement on the MOT17 dataset.

BR-YOLO and Part OSNet perform well and provide new options for multi-object detection and tracking of people in shipboard surveillance video scenarios.

## Figures and Tables

**Table 1:** Configuration information of the experimental platform.

Configuration	Versions
Operation system	Windows 10
CPU	AMD EPYC 7542 32-Core Processor
GPU	NVIDIA GeForce RTX 4090
RAM	128 GB
IDE	Pycharm2021.3.3
Compiler	Python3.8.16
Framework	Pytorch1.8.1
Toolkit	CUDA11.1 + cuDNN8.8.1

**Table 2:** Training Parameters.

Image Size	Epoch	Batch Size	Initial Learning Rate
640 × 640	500	16	0.002

**Table 3:** Evaluation indicators and trends in their optimization.

Measure	Better	Perfect
HOTA	higher	100%
MOTA	higher	100%
MOTP	higher	100%
IDF1	higher	100%
IDS	lower	0

**Table 4:** Bohai Sea Ro-Ro Ship multi-object-tracking dataset.

No.	Location	Resolution	FPS
01	Forward stairway of deck 2		
02	Forward stairway of deck 5		
03	Forward stairway of deck 9		
04	Port side of deck 10	1920 × 1080	25
05	Starboard side of deck 9		
06	Starboard side of the bridge		
07	Port side of the bridge		
08	Bridge		

**Table 5:** Results of different attention mechanisms.

Attention Mechanism	P	R	mAP0.5	mAP0.5:0.95	Param
None	0.827	0.702	0.792	0.500	**3.01 M**
CBAM	0.835	0.693	0.790	0.495	5.77 M
ECA	0.832	**0.709**	0.796	0.510	5.77 M
SE	0.829	0.698	0.791	0.497	4.26 M
GC	0.828	0.696	0.79	0.493	3.95 M
BiFormer	**0.846 **	0.702	**0.805**	**0.512**	3.08 M

**Table 6:** Results of different attention mechanisms.

Detector	P	R	mAP0.5	mAP0.5:0.95	Param	Time
YOLOv3	0.762	0.604	0.697	0.382	65.2 M	9.0
YOLOv5s	0.812	0.635	0.734	0.449	7.4 M	6.4
YOLOv7	0.838	0.720	0.799	0.512	36.72 M	7.7
YOLOv8n	0.827	0.701	0.792	0.500	3.01 M	3.8
**BR-YOLO**	0.834	0.701	0.803	0.512	2.65 M	3.8

**Table 7:** Results of object-detection ablation experiments.

Detector	Index	Modules		P	R	mAP0.5	mAP0.5:0.95	Param	Time
		BiFormer	RepGhost						
YOLOv8n	1	-	-	0.827	0.702	0.792	0.500	3.01 M	3.8
	2	✓	-	0.839	0.702	0.805	0.512	3.08 M	4.6
	3	-	✓	0.832	0.694	0.790	0.500	2.30 M	3.0
	4	✓	✓	0.834	0.701	0.803	0.512	2.65 M	3.8

**Table 8:** Comparison of tracking results among different algorithms on Bohai Sea Ro-Ro Ship Dataset.

Detector–Tracker	HOTA	MOTA	MOTP	IDF1	IDs
YOLOv8-BoT-SORT	31.608	41.704	74.859	43.330	44
YOLOv8-OC-SORT	33.168	43.391	74.708	46.049	17
YOLOv8-StrongSORT	33.564	44.162	74.869	46.569	19
YOLOv8-ByteTrack	31.134	42.658	74.828	43.203	19
YOLOv8-Deep OC-SORT	33.585	43.993	74.872	46.881	15
**Ours**	**44.243**	**53.642**	**81.295**	**58.283**	**13**

**Table 9:** Comparison of tracking results among different algorithms on MOT17 dataset.

Detector–Tracker	HOTA	MOTA	MOTP	IDF1	IDs
YOLOv8-BoT-SORT	63.449	57.371	80.004	73.732	91
YOLOv8- OC-SORT	63.452	57.248	79.993	73.716	74
YOLOv8- StrongSORT	63.586	57.264	80.261	73.266	75
YOLOv8- ByteTrack	58.381	48.771	81.233	65.753	68
YOLOv8-Deep OC-SORT	63.740	57.494	80.878	74.063	69
**Ours**	**65.385**	**58.248**	**82.229**	**76.990**	**56**

**Table 10:** Results of tracking ablation experiments.

Tracker	Index	Modules		HOTA	MOTA	MOTP	IDF1	IDs
		BR-YOLO	Part OSNet					
Deep OC-SORT	1	-	-	33.5847	43.9931	74.6716	46.8818	15
	2	✓	-	38.0400	45.9014	80.4762	47.6858	19
	3	-	✓	42.8900	52.0822	75.3015	56.0410	11
	4	✓	✓	44.2430	53.6423	81.2956	58.2834	13

## Data Availability

The dataset cannot be shared at this time as the data also form part of an ongoing study.
